# Acceptability of Bedside Resuscitation With Intact Umbilical Cord to Clinicians and Patients’ Families in the United States

**DOI:** 10.3389/fped.2018.00100

**Published:** 2018-04-26

**Authors:** Anup C. Katheria, Samuel R. Sorkhi, Kasim Hassen, Arij Faksh, Zahra Ghorishi, Debra Poeltler

**Affiliations:** Sharp Mary Birch Hospital for Women and Newborns, San Diego, CA, United States

**Keywords:** delayed cord clamping, resuscitation, survey research, obstetrics and gynecology, neonatology

## Abstract

**Background:**

While delayed umbilical cord clamping in preterm infants has shown to improve long-term neurological outcomes, infants who are thought to need resuscitation do not receive delayed cord clamping even though they may benefit the most. A mobile resuscitation platform allows infants to be resuscitated at the mother’s bedside with the cord intact. The newborn is supplied with placental blood during the resuscitation in view of the mother. The objective of the study is to assess the usability and acceptability of mobile resuscitation platform, LifeStart trolley, among the infants’ parents and perinatal providers.

**Methods:**

A resuscitation platform was present during every delivery that required advanced neonatal providers for high-risk deliveries. Perinatal providers and parents of the infants were given a questionnaire shortly after the delivery.

**Results:**

60 neonatal subjects were placed on the trolley. The majority of deliveries were high risk for meconium-stained amniotic fluid (43%), and non-reassuring fetal heart rate (45%). About 50% of neonatal providers felt that there were some concerns regarding access to the baby. No parents were uncomfortable with the bedside neonatal interventions, and most parents perceived that communication was improved because of the proximity to the care team.

**Conclusion:**

Bedside resuscitation with umbilical cord intact through the use of a mobile resuscitation trolley is feasible, safe, and effective, but about half of the perinatal providers expressed concerns. Logistical issues such as improved space management and/or delivery setup should be considered in centers planning to perform neonatal resuscitation with an intact cord.

## Introduction

In the United States, up to 10% of newborns require interventions to facilitate transition to extrauterine life while 1% need a more extensive resuscitative effort for stabilization (1). The majority of infants necessitating minor interventions undergo simple airway management, thermal care, and drying and stimulation to breathe. Infants that warrant extensive resuscitation typically require positive pressure ventilation, cardiac massage, endotracheal intubation, and drug administration (2, 3). Recently, delayed cord clamping has been shown to improve long-term neurological outcomes at 4 years of age in term infants, and a recent large RCT in preterm infants demonstrated a reduction in mortality (4). While infants that need extensive resuscitation are more likely to have higher morbidity (5), current recommendations exclude these infants from receiving delayed cord clamping primarily due to the need to quickly move the infant away to a temperature controlled resuscitation platform.

An innovative resuscitation platform, developed in the United Kingdom (LifeStart™, Inspiration HealthCare), provides heat and equipment for resuscitation while the umbilical cord is still connected to the mother (2). The arrangement is well accepted by providers and families in the United Kingdom where this practice has been in place for several years (6). We conducted a survey to evaluate the perceptions of perinatal providers and families in United States on the use of LifeStart trolley for resuscitation in singleton, high-risk vaginal deliveries.

## Materials and Methods

The LifeStart trolley resuscitation platform was first introduced at Sharp Mary Birch Hospital for Women & Newborns in 2015 as part of a preterm resuscitation trial (7). Over 200 infants had been placed on the LifeStart trolley at our institution under research protocols. In this study, we prospectively collected surveys as part of a recent trial evaluation on the use of LifeStart trolley with vaginally delivered neonates ≥37 weeks gestational age who required the attendance of advanced neonatal providers for high risk delivery. Research coordinators identified potential subjects by screening mothers in the labor and delivery unit where the attendance of advance life support team may be required. Our hospital policy mandates the attendance of an advanced life support (ALS) team for high-risk deliveries that might require instrumentation (vacuum or forceps assisted vaginal delivery), or presentation with meconium-stained amniotic fluid, or non-reassuring fetal heart rate. All infants were either placed on the resuscitation platform or the mother’s abdomen, with the equipment on the trolley used to provide resuscitation. The Sharp Health Care Institutional Review Board approved the study. Written informed consent was obtained by research staff prior to delivery for all infants. This study was conducted over a 2-month period from August to September 2016. Complications requiring cesarean sections, congenital anomalies, and mothers with multiple gestations were excluded.

Details on the neonatal resuscitation interventions including the need for stimulation to breathe, positive pressure ventilation, requirement of intubation, chest compressions, and administration of epinephrine were recorded as part of a randomized controlled trial of delayed cord clamping in term newborns at risk for resuscitation (8). Shortly after the delivery, de-identified questionnaires in envelopes were distributed to perinatal and neonatal providers and the infants’ parents to assess the usability and acceptability of bedside resuscitation with intact umbilical cord. Each questionnaire consisted of both closed and open-ended questions. Parents and clinicians were instructed to answer the questionnaires in private and return the completed questionnaires in a sealed envelope. The parents responded to questions regarding overall satisfaction and their ability to see or touch their infant. The clinician questionnaire included questions regarding barriers encountered during resuscitation, perceived impediments to care, and the effect that bedside resuscitation has on the mother’s and baby’s health. The surveys were collected within 2–12 h after distribution.

## Results

The demographics of the 60 neonatal subjects and the indications for ALS team involvement are provided in Table 1. Demographics of the infants and mother have also been previously reported (8). The majority of deliveries were deemed high risk for meconium-stained amniotic fluid (43%), followed by non-reassuring fetal heart rate (45%). Thirty of the infants initially were placed on the trolley and 48 were initially or eventually, after a brief evaluation on the trolley, placed on the mother’s abdomen. The interventions performed on the trolley or abdomen are shown in Table 2. No infant had an umbilical cord too short to be placed on the trolley with the exception of an infant with a nuchal cord that was difficult to reduce and another who experienced cord avulsion when attempting to place on the trolley.

**Table 1:** Neonatal demographics.

Demographics	*n* = 60
Gestational age (weeks), mean (±SD)	39 ± 1.0
Male, *n* (%)	33 (55)
Birth weight (g), mean (± SD)	3,385 ± 511
1 min Apgar, median [IQR]	8 [8,8]
5 min Apgar, median [IQR]	9 [9,9]
1 min Apgar ≤ 7, *n* (%)	12 (20.0)
5 min Apgar ≤ 7, *n* (%)	2 (3.3)
Non-reassuring fetal HR and meconium-stained amniotic fluid, *n* (%)	12 (20)
Non-reassuring fetal HR only, *n* (%)	15 (25.0)
Meconium-stained amniotic fluid only, *n* (%)	26 (43.33)
Others^a^, *n* (%)	7 (11.67)
Admitted to NICU, *n* (%)	8 (13.3)

*^a^One instrumentation, one preeclampsia, two shoulder dystocia, one CCAM, one fetal tachycardia, and one no prenatal care*.

**Table 2:** Neonatal interventions.

Highest level of support required	*n* = 60
No support, *n* (%)	28 (46.7)
Stimulation only, *n* (%)	8 (13.3)
Supplemental oxygen, *n* (%)	15 (25.0)
Oral/nasal suction (catheter or meconium aspirator), *n* (%)	1 (1.7)
CPAP, *n* (%)	1 (1.7)
Positive pressure ventilation, *n* (%)	5 (8.3)
Required intubation, *n* (%)	2 (3.3)
Required chest compression or epinephrine, *n* (%)	0

A total of 167 responses were collected. A summary of the respondents is shown in Table 3. The provider’s responses to the questionnaire are shown in Table 4. About 50% of neonatal providers, including obstetricians, expressed some concerns regarding suboptimal access to the baby. Two clinicians felt “strongly negative” about resuscitation at bedside, which may have been due to the infant requiring intubation while on the trolley. The parent’s responses are shown in Table 5. Most parents were able to see and make contact with their child in the first few minutes of life. All parents had positive impression of both resuscitation at bedside and on a radiant warmer. No parent felt uncomfortable with neonatal interventions being provided at bedside. They also perceived that their close proximity improved communication.

**Table 3:** Survey respondents.

Respondents	*n* = (167)
Obstetricians	11
Labor and delivery nurse	38
Advanced life support (ALS) nurse	36
ALS respiratory therapist	25
Parents	57

**Table 4:** Responses to the Provider Questionnaire (obstetrical and neonatal).

**How did the trolley or resuscitation on the mom compare with moving the infant to a standard radiant warmer? (*n* = 110)**						

Short cord			21			
Poor access to the baby			17			
Difficulty accessing equipment			14			
Uncomfortable with parents watching			9			

**Did the resuscitation on the cord impede your care?**						

	**Not at all**	**Slightly**	**Moderately**	**Very**	**Extremely**	**N/A**

Neonatal providers (*n* = 61)	29 (47.5)	17 (27.9)	4 (6.6)	2 (3.3)	2 (3.3)	29 (47.5)
Maternal providers (*n* = 49)	26 (53.06)	19 (38.8)	7 (14.3)	1 (2.0)	0	0

**How would you characterize the process of providing the baby’s resuscitation at the mother’s bedside?**						

	**Strongly negative**	**Negative**	**Neutral**	**Positive**	**Strongly positive**	**NA**

Neonatal provider (*n* = 61)	2 (3.3)	5 (8.2)	15 (24.6)	19 (31.1)	12 (19.7)	6 (9.8)
Maternal providers (*n* = 49)	0	3 (6.1)	9 (18.4)	14 (28.6)	16 (32.7)	3 (6.1)

**How would you characterize utilizing the LifeStart bed as a platform for resuscitation compared to using the radiant warmer?**						

	**Strongly negative**	**Negative**	**Neutral**	**Positive**	**Strongly positive**	**NA**

Neonatal providers (*n* = 61)	2 (2.3)	7 (11.5)	16 (26.2)	11 (18.0)	7 (11.5)	13 (21.3)
Maternal providers (*n* = 49)	0	1 (2.0)	9 (18.4)	16 (32.7)	8 (16.3)	12 (24.5)

**Table 5:** Responses to the parent questionnaire.

**Given my baby’s participation in this research study, would you consider participation in another research study in the future?**					

**Strongly negative**	**Negative**	**Neutral**	**Positive**		**Strongly positive**

0	0	7 (12.3)	27 (47.4)		23 (40.4)

**What impact do you feel participation in this study had on your baby’s health?**					

**Strongly negative**	**Negative**	**Neutral**	**Positive**		**Strongly positive**

0	0	12 (21)	24 (42.1)		21 (36.8)

**How much visual contact did you have with your baby immediately after birth?**					

**Never**	**Rarely**	**Sometimes**	**Often**		**Always**

1 (1.75)	1 (1.75)	4 (7)	13 (22.8)		38 (66.7)

**How much were you able to touch your baby immediately after birth?**					

**Never**	**Rarely**	**Sometimes**	**Often**		**Always**

1 (1.75)	2 (3.5)	8 (14)	14 (24.6)		32 (56.1)

**How comfortable were you with the baby’s care occurring across the room on the warming bed?**					

**Not comfortable**	**Slightly comfortable**	**Moderately comfortable**	**Very comfortable**	**Completely comfortable**	**N/A**

0	2 (3.7)	4 (7.4)	12 (22.2)	13 (24)	23 (42.6)

**How comfortable were you with your baby’s care occurring on a small bed next to mother?**					

**Not comfortable**	**Slightly comfortable**	**Moderately comfortable**	**Very comfortable**	**Completely comfortable**	**N/A**

0	0	2 (3.8)	12 (22.6)	12 (22.6)	27 (50.9)

## Discussion

Currently, when a resuscitation is required, the infant is immediately moved to a resuscitation area at a fixed distance away from the mother. This separation leaves parents in a high level of anxiety and stress (9). A large multicenter randomized controlled trial in the United Kingdom, where 137 preterm infants were randomized to resuscitated with intact cord when needed versus immediate cord clamping, reported a decreased mortality with delayed cord clamping (10). They also reported no difference in compliance between sites that used traditional resuscitation equipment and trolley.

This survey, to our knowledge, is the first to report both parental and provider assessments of performing resuscitation at the maternal bedside in the United States. There was a substantial percentage of neonatal (35%) and perinatal providers (24%) who felt that the trolley had some adverse impact on the delivery of care. The results of our survey are slightly different to those reported by Thomas et al. (2). In their study, the authors evaluated both term and premature newborns as well as those delivered by cesarean section. The majority of clinicians (86%) in their study rated the resuscitation trolley as the “same,” “better,” or “much better” than conventional equipment.

In our survey, 41% of neonatal providers and 32% of maternal providers characterized the process of resuscitation on the LifeStart trolley compared to the traditional resuscitation platform as positive or strongly positive for the infant. While there were no actual cases where the cord was too short for the baby to be placed on the trolley, about one-third said that the cord was felt to be too short and had poor access to the baby. Twenty-five percentage also felt that they had difficulty accessing equipment.

The majority of parents felt that the experience of bedside resuscitation was positive possibly because they were able to visualize and or touch their newborn immediately after delivery and throughout resuscitation; however, a number of neonatal and maternal providers (16%) felt uncomfortable with the family being present at the resuscitation.

This study has a number of limitations. The survey was part of a research study and the sample only included infants consented to that study (8). The study was evaluating delayed umbilical cord clamping for ≥5 min versus immediate cord clamping in term infants. Since delayed cord clamping for this duration has not been well studied especially regarding maternal outcomes (hemorrhage etc.), only vaginal deliveries were included. Several of the consented mothers went on to have a cesarean section for further deterioration and many of those infants would have likely been sicker than our cohort. However, vaginal deliveries allowed for the parents to interact with infant at birth and evaluate parental perceptions of the stabilization process.

**Figure 1:**
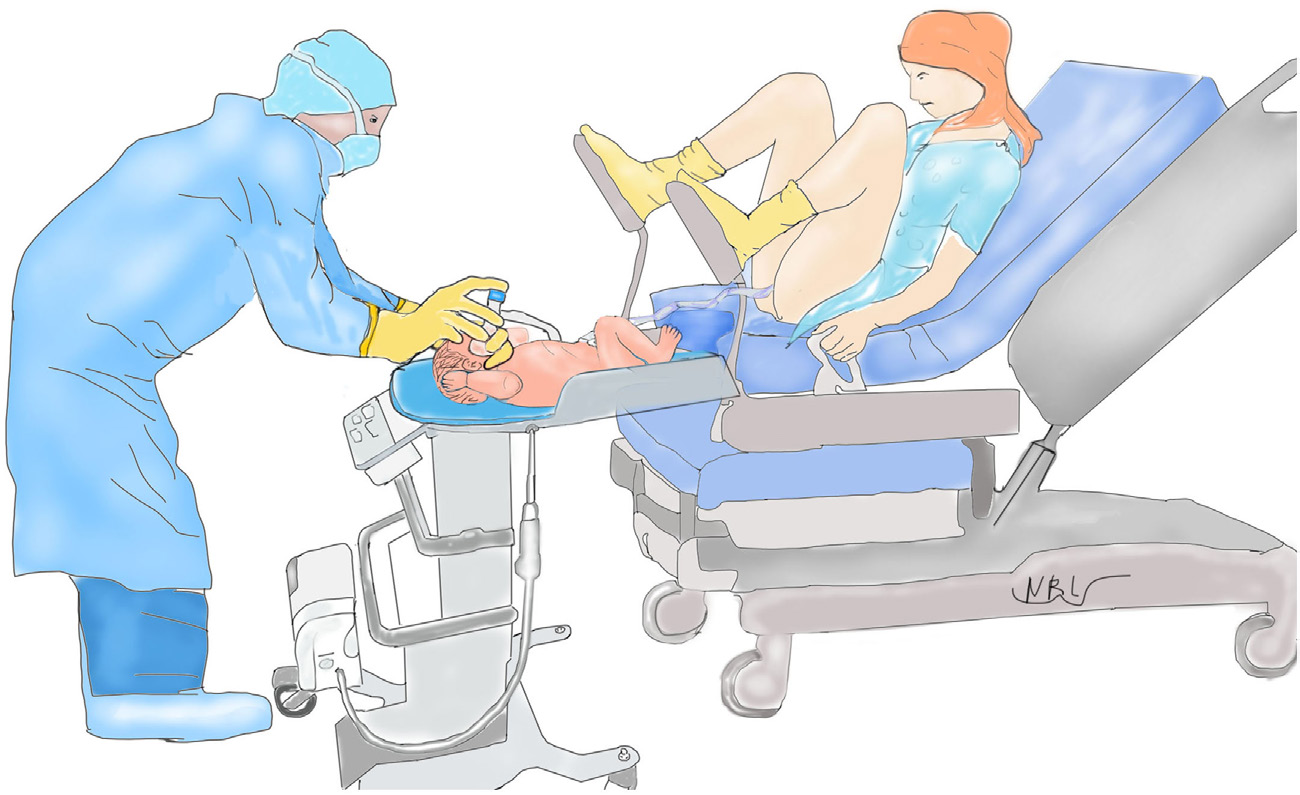
Setup for vaginal delivery in the United States.

**Figure 2:**
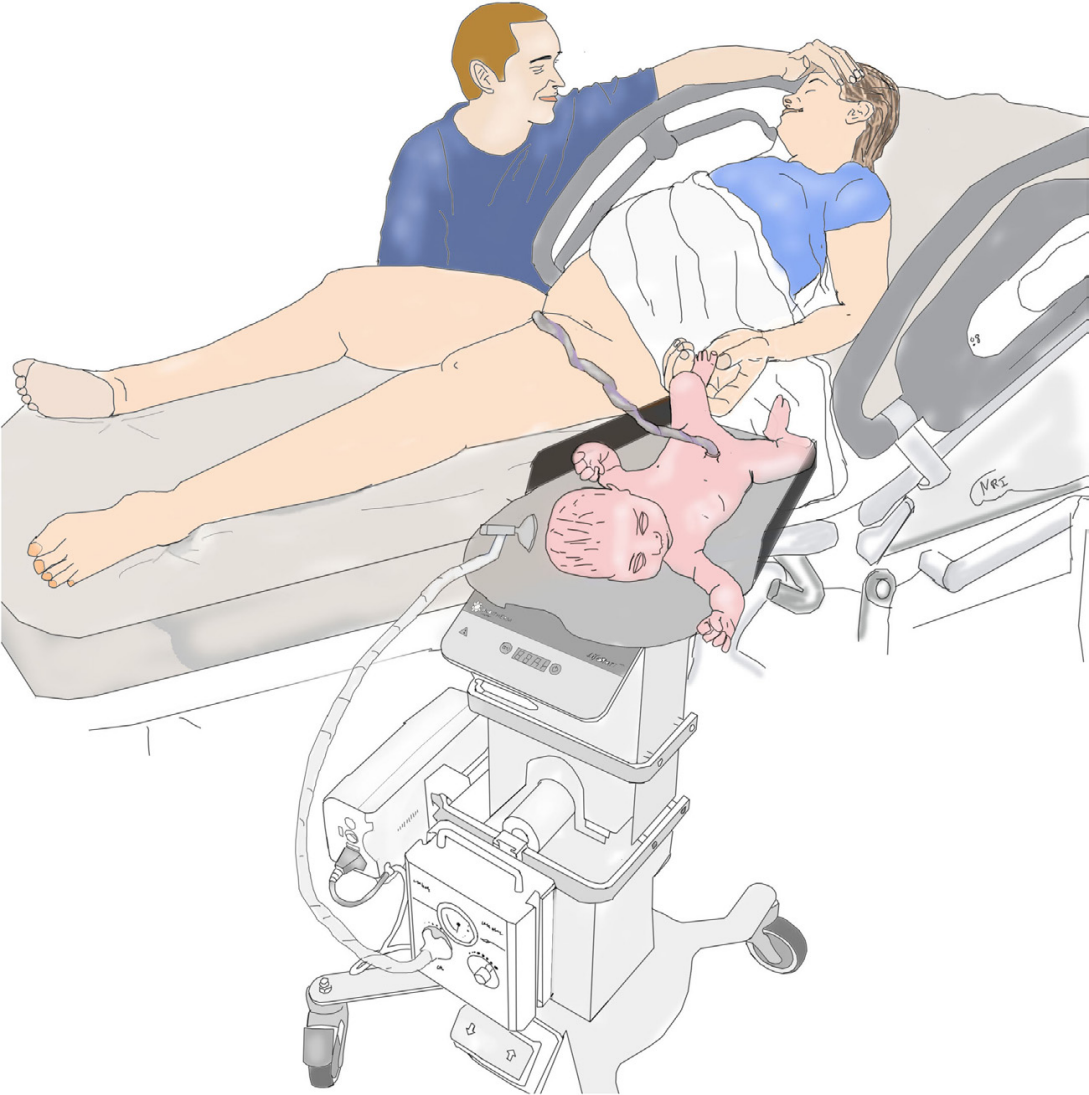
Setup for vaginal delivery in the United Kingdom.

Our results may not be generalizable to other institutions or countries because of major institutional differences and study limitations. At our institution vaginal deliveries are performed exclusively by obstetricians with delivery beds consistently being broken down and women placed in lithotomy position. This maternal positioning creates enough space for LifeStart trolley to only be placed close enough to the vagina (see Figure 1). In the United Kingdom, vaginal deliveries occur without the beds being broken down allowing for the infant to be placed next to the mother (see Figure 2).

We have previously shown the physiological benefits of resuscitation (higher cerebral oxygenation and blood pressure) with an intact cord in term infants (8). However, in the United States further change in the obstetrical and neonatal culture is needed before the resuscitation with an intact cord can be universally adopted. As the practice becomes widespread, logistical issues such as spacing of personnel and availability of equipment must be addressed. Hospitals considering the purchase and use of this equipment must ensure that the staff and clinicians are properly trained and educated to facilitate a smooth transition.

It is reassuring that families in the United States also view resuscitation with an intact cord as a positive experience. Fortunately, we did not have any infants receive extensive resuscitation measures, but future studies should account for this. Based on prior literature, families appreciate seeing their child receive all the treatment despite unexpected clinical outcomes (9, 11, 12). While a few neonatal and obstetrical providers did acknowledge being uncomfortable being in parents’ view, we believe this could be overcome with more experience.

## Conclusion

This study demonstrates that neonatal resuscitation during delayed cord clamping is favored by parents and clinicians; however, there are some logistical considerations that need better evaluation prior to the widespread adoption of this intervention. Improved space and access issues must be resolved, particularly, if more extensive resuscitation is anticipated. Overall, families in this study felt that bedside resuscitation was a positive experience. Further studies should include more diverse demographics, infants requiring intensive resuscitations, and evaluation of clinician and parental perspectives under broader clinical circumstances.

## Ethics Statement

This study was carried out in accordance with the recommendations of Sharp HealthCare Institutional Review board. The protocol was approved by the Sharp HealthCare IRB. All subjects gave written informed consent in accordance with the Declaration of Helsinki.

## Author Contributions

AK conceived the study and helped to write the initial draft. SS helped to write the initial draft and collect the data. KH contributed on revisions of the initial draft and helped interpret the data AF worked with the obstetricians to collect data and perform the interventions and edited the manuscript. ZG contributed to the revision of the manuscript. DP performed statistical analysis for the data and helped with the results section of the manuscript.

## Conflict of Interest Statement

The authors declare that the research was conducted in the absence of any commercial or financial relationships that could be construed as a potential conflict of interest.
